# Supplementary figures and images for: Analysis of the earliest complete mtDNA genome of a Caribbean colonial horse (Equus caballus) from 16th-century Haiti
Source: PLoS One. 2022 Jul 27;17(7):e0270600. doi: 10.1371/journal.pone.0270600 (PMC9328532; doi:10.1371/journal.pone.0270600)

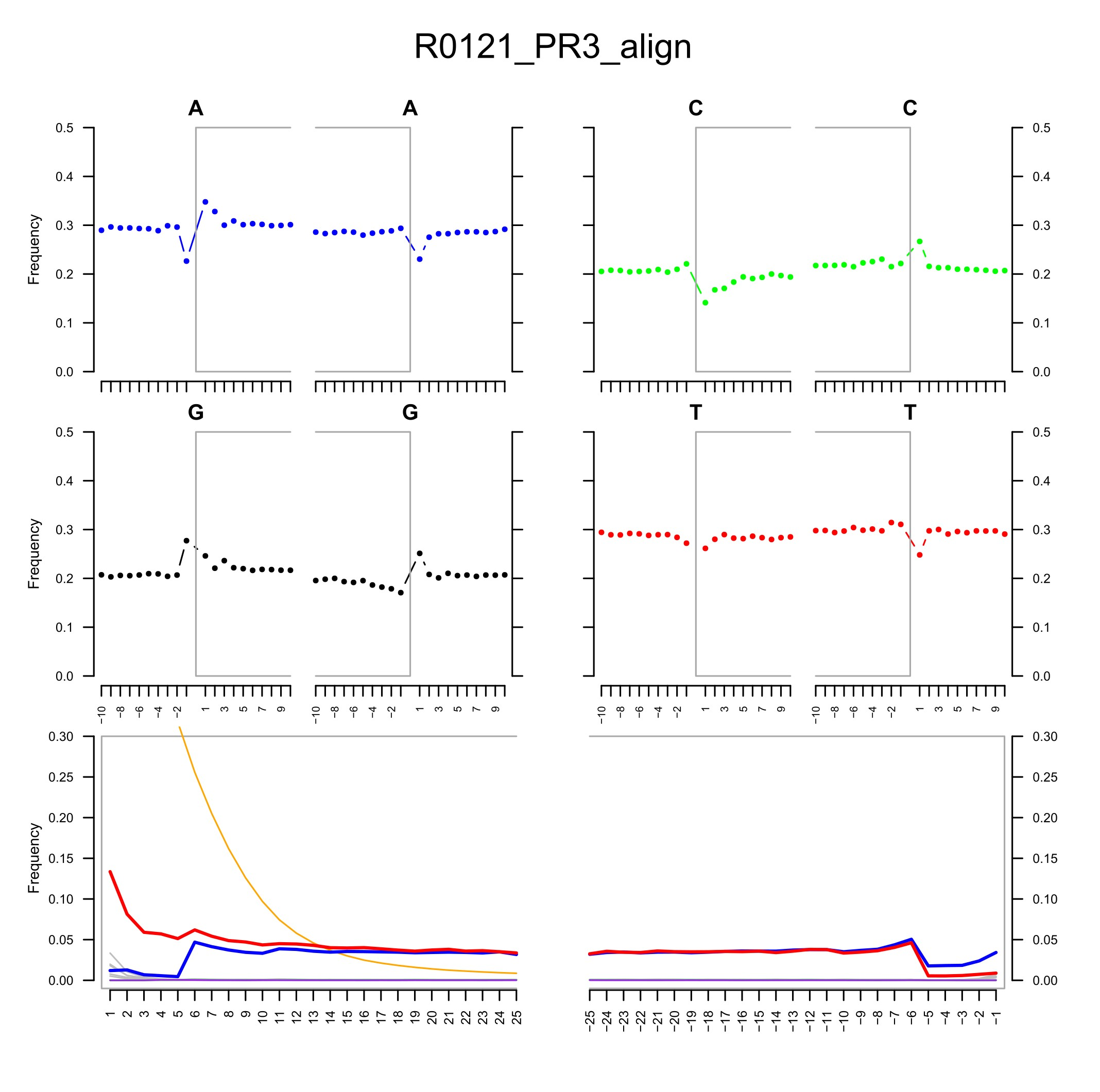

Supplement: S1 Fig — (TIF) [file pone.0270600.s001.tif]

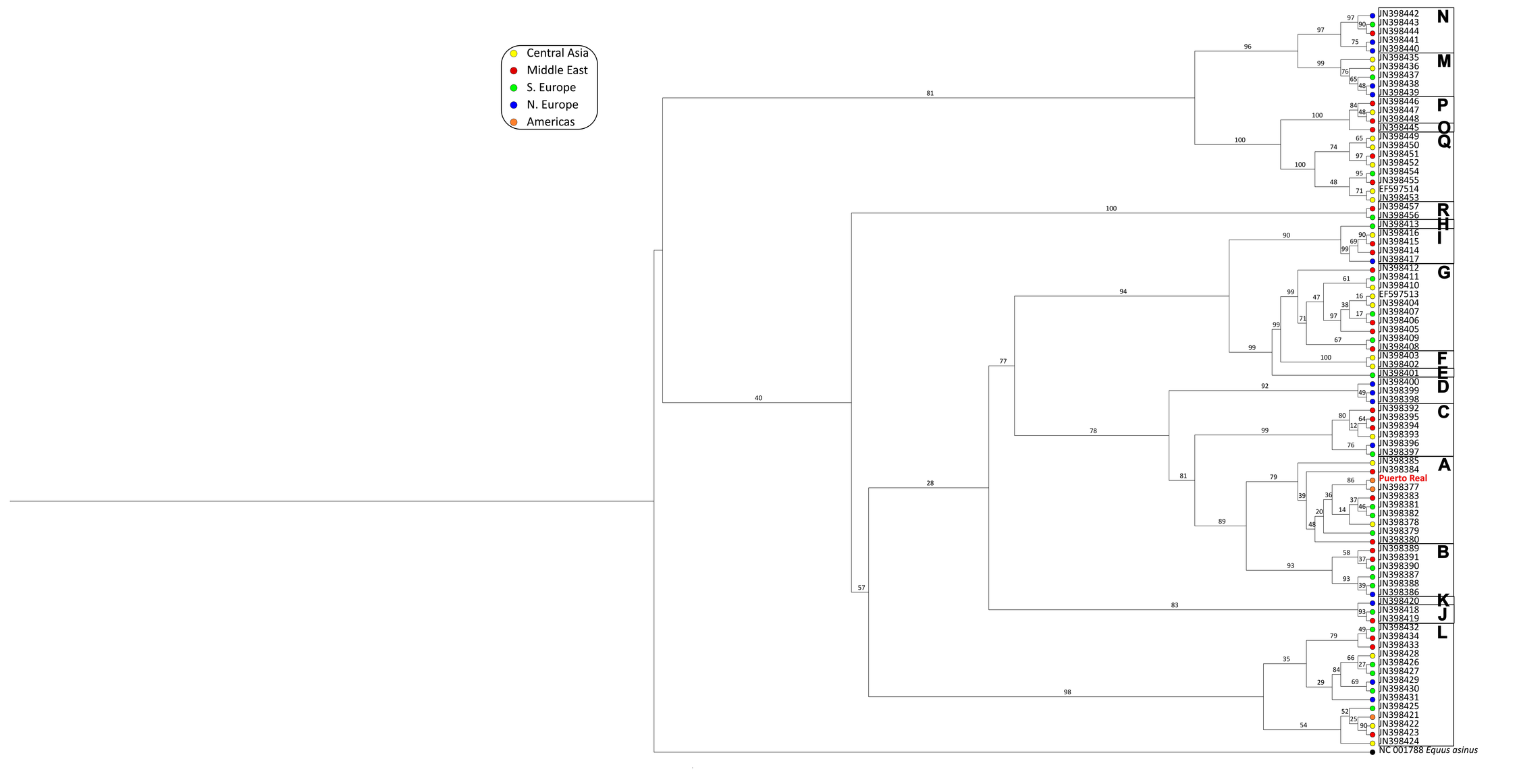

Supplement: S2 Fig — Bootstrap support is on the nodes (10,000 iterations). Letters on the right correspond to the equine haplogroups defined by Achilli et al. [22]. (TIF) [file pone.0270600.s002.tif]
