# Supplementary material for: Analysis of the earliest complete mtDNA genome of a Caribbean colonial horse (Equus caballus) from 16th-century Haiti
Source: PLoS One. 2022 Jul 27;17(7):e0270600. doi: 10.1371/journal.pone.0270600 (PMC9328532; doi:10.1371/journal.pone.0270600)
Supplement: S1 Table — Except for NC001788 (Equus asinus) all are published in Achilli et al. [22]. (DOCX) [file pone.0270600.s003.docx]

| GenBank Accession # | Species | Breed | Haplogroup |
| --- | --- | --- | --- |
| JN398377 | *Equus caballus* | Chincoteague Pony | A |
| JN398379 | *Equus caballus* | Maremmano | A |
| JN398383 | *Equus caballus* | unspecified Iranian Breed | A |
| JN398381 | *Equus caballus* | Maremmano | A |
| JN398380 | *Equus caballus* | Arabian | A |
| JN398385 | *Equus caballus* | Akhal-Teke | A |
| JN398384 | *Equus caballus* | unspecified Syrian Breed | A |
| JN398378 | *Equus caballus* | Caspian Pony | A |
| JN398382 | *Equus caballus* | Maremmano | A |
| JN398390 | *Equus caballus* | unspecified Italian Breed | B |
| JN398391 | *Equus caballus* | unspecified Syrian Breed | B |
| JN398389 | *Equus caballus* | unspecified Syrian Breed | B |
| JN398386 | *Equus caballus* | Westphalian | B |
| JN398388 | *Equus caballus* | Maremmano | B |
| JN398387 | *Equus caballus* | Maremmano | B |
| JN398396 | *Equus caballus* | Suffolk Punch | C |
| JN398395 | *Equus caballus* | unspecified Iranian Breed | C |
| JN398397 | *Equus caballus* | Maremmano | C |
| JN398394 | *Equus caballus* | unspecified Syrian Breed | C |
| JN398392 | *Equus caballus* | Arabian | C |
| JN398393 | *Equus caballus* | Akhal-Teke | C |
| JN398398 | *Equus caballus* | Norwegian Fjord | D |
| JN398399 | *Equus caballus* | Icelandic Horse | D |
| JN398400 | *Equus caballus* | Icelandic Horse | D |
| JN398401 | *Equus caballus* | Maremmano | E |
| JN398402 | *Equus caballus* | Przewalskii | F |
| JN398403 | *Equus caballus* | Przewalskii | F |
| JN398410 | *Equus caballus* | Akhal-Teke | G |
| JN398411 | *Equus caballus* | Giara Horse (Sardinia) | G |
| JN398412 | *Equus caballus* | Arabian | G |
| JN398408 | *Equus caballus* | unspecified Syrian Breed | G |
| JN398409 | *Equus caballus* | unspecified Italian Breed | G |
| JN398405 | *Equus caballus* | unspecified Iranian Breed | G |
| JN398407 | *Equus caballus* | Giara Horse (Sardinia) | G |
| EF597513 | *Equus caballus* | Naqu | G |
| JN398404 | *Equus caballus* | Akhal-Teke | G |
| JN398406 | *Equus caballus* | Arabian | G |
| JN398413 | *Equus caballus* | Maremmano | H |
| JN398414 | *Equus caballus* | unspecified Iranian Breed | I |
| JN398417 | *Equus caballus* | Trakhener | I |
| JN398415 | *Equus caballus* | unspecified Iranian Breed | I |
| JN398416 | *Equus caballus* | Caspian Pony | I |
| JN398418 | *Equus caballus* | Maremmano | J |
| JN398419 | *Equus caballus* | unspecified Iranian Breed | J |
| JN398420 | *Equus caballus* | Belgian Draft | K |
| JN398433 | *Equus caballus* | unspecified Iranian Breed | L |
| JN398432 | *Equus caballus* | Maremmano | L |
| JN398434 | *Equus caballus* | Arabian | L |
| JN398424 | *Equus caballus* | Akhal-Teke | L |
| JN398422 | *Equus caballus* | Akhal-Teke | L |
| JN398423 | *Equus caballus* | unspecified Iranian Breed | L |
| JN398421 | *Equus caballus* | American Paint Horse | L |
| JN398425 | *Equus caballus* | unspecified Italian Breed | L |
| JN398426 | *Equus caballus* | Maremmano | L |
| JN398429 | *Equus caballus* | Oldenburg | L |
| JN398431 | *Equus caballus* | Silesian | L |
| JN398427 | *Equus caballus* | Maremmano | L |
| JN398428 | *Equus caballus* | Caspian Pony | L |
| JN398430 | *Equus caballus* | Andalusian | L |
| JN398435 | *Equus caballus* | Akhal-Teke | M |
| JN398438 | *Equus caballus* | Friesian | M |
| JN398436 | *Equus caballus* | Caspian Pony | M |
| JN398437 | *Equus caballus* | Maremmano | M |
| JN398439 | *Equus caballus* | Clydesdale | M |
| JN398440 | *Equus caballus* | English Shire | N |
| JN398441 | *Equus caballus* | Saddlebred | N |
| JN398442 | *Equus caballus* | Exmoor Pony | N |
| JN398443 | *Equus caballus* | Andalusian | N |
| JN398444 | *Equus caballus* | unspecified Iranian Breed | N |
| JN398445 | *Equus caballus* | unspecified Iranian Breed | O |
| JN398448 | *Equus caballus* | Arabian | P |
| JN398446 | *Equus caballus* | unspecified Iranian Breed | P |
| JN398447 | *Equus caballus* | Caspian Pony | P |
| EF597514 | *Equus caballus* | Deqin | Q |
| JN398453 | *Equus caballus* | Akhal-Teke | Q |
| JN398454 | *Equus caballus* | Maremmano | Q |
| JN398455 | *Equus caballus* | unspecified Iranian Breed | Q |
| JN398451 | *Equus caballus* | unspecified Iranian Breed | Q |
| JN398452 | *Equus caballus* | Akhal-Teke | Q |
| JN398449 | *Equus caballus* | Akhal-Teke | Q |
| JN398450 | *Equus caballus* | Akhal-Teke | Q |
| JN398456 | *Equus caballus* | Maremmano | R |
| JN398457 | *Equus caballus* | unspecified Iranian Breed | R |
| NC001788 | *Equus asinus* |  |  |
