# Supplementary material for: Analysis of the earliest complete mtDNA genome of a Caribbean colonial horse (Equus caballus) from 16th-century Haiti
Source: PLoS One. 2022 Jul 27;17(7):e0270600. doi: 10.1371/journal.pone.0270600 (PMC9328532; doi:10.1371/journal.pone.0270600)
Supplement: Attachment — Submitted filename: PLOS1 rebuttal letter_Delsol_et_al_final.docx [file pone.0270600.s005.docx]

Nicolas Delsol

Florida Museum of Natural History - Environmental Archaeology program

1659 Museum Road

Gainesville, FL 32611 – USA

[ndelsol@ufl.edu](mailto:ndelsol@ufl.edu)

Gainesville, April 10, 2022

To the PLOS One editing committee,

We would like to thank the academic editor and the reviewers for their thoughtful comments which have greatly helped improve our manuscript. Please find here our response to the points they raised. For clarity purposes, the editor and reviewers’ initial comments are inserted in *blue italic* font before our responses.

**A. Editor’s comment:**

1. *Please ensure that your manuscript meets PLOS ONE's style requirements, including those for file naming. The PLOS ONE style templates can be found at*

*https://journals.plos.org/plosone/s/file?id=wjVg/PLOSOne_formatting_sample_main_body.pdf and*

*https://journals.plos.org/plosone/s/file?id=ba62/PLOSOne_formatting_sample_title_authors_affiliations.pdf*

We made sure to implement PLOS ONE’s style template in the main manuscript and changed the figures file names to follow the file naming template.

2. *In your manuscript, please provide additional information regarding the specimens used in your study. Ensure that you have reported specimen numbers and complete repository information, including museum name and geographic location.*

*If permits were required, please ensure that you have provided details for all permits that were obtained, including the full name of the issuing authority, and add the following statement:*

*'All necessary permits were obtained for the described study, which complied with all relevant regulations.'*

*If no permits were required, please include the following statement:*

*'No permits were required for the described study, which complied with all relevant regulations.'*

*For more information on PLOS ONE's requirements for paleontology and archaeology research, see https://journals.plos.org/plosone/s/submission-guidelines#loc-paleontology-and-archaeology-research.*

We added a Methods subsection that details additional information on the permits and curation institution, including the statement 'All necessary permits were obtained for the described study, which complied with all relevant regulations.'

3. *Please include a complete copy of PLOS’ questionnaire on inclusiveness in global research in your revised manuscript. Our policy for research in this area aims to improve transparency in the reporting of research performed outside of researchers’ own country or community. The policy applies to researchers who have traveled to a different country to conduct research, research with Indigenous populations or their lands, and research on cultural artifacts. The questionnaire can also be requested at the journal’s discretion for any other submissions, even if these conditions are not met. Please find more information on the policy and a link to download a blank copy of the questionnaire here: https://journals.plos.org/plosone/s/best-practices-in-research-reporting. Please upload a completed version of your questionnaire as Supporting Information when you resubmit your manuscript.*

The questionnaire on inclusiveness in global research will be uploaded in the SI section together with the revised documents.

4. *We note that Figure 1 in your submission contain [map/satellite] images which may be copyrighted. All PLOS content is published under the Creative Commons Attribution License (CC BY 4.0), which means that the manuscript, images, and Supporting Information files will be freely available online, and any third party is permitted to access, download, copy, distribute, and use these materials in any way, even commercially, with proper attribution. For these reasons, we cannot publish previously copyrighted maps or satellite images created using proprietary data, such as Google software (Google Maps, Street View, and Earth). For more information, see our copyright guidelines: http://journals.plos.org/plosone/s/licenses-and-copyright.*

*We require you to either (1) present written permission from the copyright holder to publish these figures specifically under the CC BY 4.0 license, or (2) remove the figures from your submission:*

*1. You may seek permission from the original copyright holder of Figures 1 to publish the content specifically under the CC BY 4.0 license.*

*We recommend that you contact the original copyright holder with the Content Permission Form (http://journals.plos.org/plosone/s/file?id=7c09/content-permission-form.pdf) and the following text:*

*“I request permission for the open-access journal PLOS ONE to publish XXX under the Creative Commons Attribution License (CCAL) CC BY 4.0 (http://creativecommons.org/licenses/by/4.0/). Please be aware that this license allows unrestricted use and distribution, even commercially, by third parties. Please reply and provide explicit written permission to publish XXX under a CC BY license and complete the attached form.”*

*Please upload the completed Content Permission Form or other proof of granted permissions as an "Other" file with your submission.*

*In the figure caption of the copyrighted figure, please include the following text: “Reprinted from [ref] under a CC BY license, with permission from [name of publisher], original copyright [original copyright year].”*

*2. If you are unable to obtain permission from the original copyright holder to publish these figures under the CC BY 4.0 license or if the copyright holder’s requirements are incompatible with the CC BY 4.0 license, please either i) remove the figure or ii) supply a replacement figure that complies with the CC BY 4.0 license. Please check copyright information on all replacement figures and update the figure caption with source information. If applicable, please specify in the figure caption text when a figure is similar but not identical to the original image and is therefore for illustrative purposes only.*

*The following resources for replacing copyrighted map figures may be helpful:*

*USGS National Map Viewer (public domain): http://viewer.nationalmap.gov/viewer/*

*The Gateway to Astronaut Photography of Earth (public domain): http://eol.jsc.nasa.gov/sseop/clickmap/*

*Maps at the CIA (public domain): https://www.cia.gov/library/publications/the-world- factbook/index.html and https://www.cia.gov/library/publications/cia-maps-publications/index.html*

*NASA Earth Observatory (public domain): http://earthobservatory.nasa.gov/*

*Landsat: http://landsat.visibleearth.nasa.gov/*

*USGS EROS (Earth Resources Observatory and Science (EROS) Center) (public domain): http://eros.usgs.gov/#*

Figure 1 is a map that was designed by the lead author (Nicolas Delsol) using the open-source GIS software QGIS (version 3.24.1). The rasters used for the design of this map come from public and non-copyright cartographic resources generated by the NASA and and Japan’s Ministry of Economy, Trade, and Industry (METI). These resources were retrieved at the url [http://eros.usgs.gov/#](https://urldefense.proofpoint.com/v2/url?u=http-3A__eros.usgs.gov_&d=DwMGaQ&c=sJ6xIWYx-zLMB3EPkvcnVg&r=7ytBbmhjXOarCt74HUuf9g&m=E5bPX627S2ySm2XbrRC1UUne-fU8odbmNtXXoev6Dt3-EeqF5cmqF72wOZD51yub&s=RQ1hThlkXPBtQh9yFyEz_69EGVIwXPl43bgepHjpXO8&e=)  and were recommended by the Academic Editor in his comments. In the caption of the figure we mention the source of this raw cartographic data.

5. *Thank you for stating the following in the Acknowledgments Section of your manuscript:*

*“This research was funded by the National Science Foundation to Emery and Delsol (DDIG 1930628), the Fulbright program (to Delsol), and additional support from the Florida Museum Ancient DNA Laboratory (from Guralnick). Permission for excavation and export of the faunal materials for curation at the Florida Museum was provided to Deagan by the government of Haiti, and permission for analysis was provided by the curators of the Historical Archaeology (Cobb) and Environmental Archaeology (Emery) programs of the Florida Museum.”*

*We note that you have provided additional information within the Acknowledgements Section that is not currently declared in your Funding Statement. Please note that funding information should not appear in the Acknowledgments section or other areas of your manuscript. We will only publish funding information present in the Funding Statement section of the online submission form.*

*Please remove any funding-related text from the manuscript and let us know how you would like to update your Funding Statement. Currently, your Funding Statement reads as follows:*

*“KFE and ND received support from NSF DDRIG grant (#1930628) for this research (https://beta.nsf.gov/funding/opportunities/archaeology-and-archaeometry-0). The funders had no role in study design, data collection and analysis, decision to publish, or preparation of the manuscript.”*

*Please include your amended statements within your cover letter; we will change the online submission form on your behalf.*

The Acknowledgement section has been changed to reflect the Editor’s requests. We also removed funding-related text from the manuscript.

In addition, we ask the Academic Editor to change the funding statement in the following way:

“KFE and ND received support from NSF DDRIG grant (#1930628) for this research ([https://beta.nsf.gov/funding/opportunities/archaeology-and-archaeometry-0](https://urldefense.proofpoint.com/v2/url?u=https-3A__beta.nsf.gov_funding_opportunities_archaeology-2Dand-2Darchaeometry-2D0&d=DwQGaQ&c=sJ6xIWYx-zLMB3EPkvcnVg&r=7ytBbmhjXOarCt74HUuf9g&m=E5bPX627S2ySm2XbrRC1UUne-fU8odbmNtXXoev6Dt3-EeqF5cmqF72wOZD51yub&s=t9jftuulFEwISTizbSlFv9r3voQegRI41QpxfAVaPEA&e=)). ND’s initial research was also funded by a Fulbright scholarship. The field research in Haiti was financed through contributions of the Organization of American States, the National Endowment for the Humanities, Dr. William Goya, the Wentworth Foundation, the University of Florida Division of Sponsored Research, and the Florida Museum of Natural History. The funders had no role in study design, data collection and analysis, decision to publish, or preparation of the manuscript.”

**B. Reviewers’ comments:**

- Reviewers #2, #3, and #4 expressed some concern about the availability of the consensus sequence and the raw reads on public databases. The raw reads have been deposited on the NCBI SRA repository (BioProject# PRJNA817535, SRA# SRR18395639). The annotated mitochondrial consensus sequence has been deposited on Genbank under the reference # ON168403. Both datasets are set to be publicly released upon publication of the article.
- **Reviewer #2:**

*The manuscript reads very well and presents a nice story that fits with the historical records for the introduction of the horse into the Americas in the 16th century. The only minor issue with the results presented is that it is only based on a single specimen.*

*While the authors indicate that the sequence is publicly available at the NCBI, no accession number is provided. The accession number needs to be added before the paper can be published.*

*For the rest, I only have a few minor comments:*

*Line 73: "By the 1650s century" delete "century"*

*Lines 167-175. It would have been nice if the authors would have added a map of the archeological site in the supplementary material.*

*Lines 232-237: I assume the medium-joining network mentioned in this paragraph is the one shown in figure 3?*

*Line 259: "Figure S1" should be "Figure S2"*

We made the minor revisions asked for on lines 73 and 259. We also added a reference to Fig 3 to refer to the median-joining network. About the site map, we did not have access to a high-quality reproduction of a map of the site, given that most publications on this site are almost 30 years old. The syntheses of the site that were cited (Deagan 1995, 1996) provide some cartographic data.

- **Reviewer #3:**

*The paper reports assembly of the whole mt genome of a single horse dating back to 16th century. It is well written, and although only one sample was analyzed I think it adds to current knowledge.*

*However, I have several suggestions to improve the paper before publication on PLOS ONE, mainly related to reproducibility and data availability.*

*In particular, authors declared that they made the data publicly available, but I wasn't able to find any link pointing to them.*

*I list my concerns below regarding Data availability and Methods (and reproducibility)*

*Data availability issues:*

*Raw reads should be deposited on SRA. The assembled genome should be deposited in GenBank. Authors declared that the data are available, but they are not.*

*Methods and reproducibility issues:*

*First of all, I think authors performed all the analysis (mapping, assembling, phylogenetic tree construction) using the Geneious commercial software. This should be clearly declared.*

*Most importantly, for each analysis step, all the parameters and options used should be clarified so that a user buying Genious and having access to the data would be able to repeat the experiment. I think we are still far from this. I give some suggestions below, but I invite the authors to carefully check the methods and identify more missing details.*

*Line 210: “The 3’ and 5’ reads were imported, paired, and deduplicated in Geneious (https://www.geneious.com/).” This description should be more detailed. 1) What does it mean that read were “imported, paired”? Reads are already provided as paired by the sequencing instrument. Did the authors mean that the tried to merge paired reads in a single read? 2) What does it mean that reads were “deduplicated”? Did authors remove duplicates? 3) I assume 3’ and 5’ reads are the so-called forward and reverse reads. I think it is more common to name them forward and reverse, but this is not an issue.*

*Line 211: “The sequence was attributed to a horse after a BLAST search on the reads.” Did the authors use the blast online service? If so, they should provide the link and/or a reference to BLAST publications. Did the authors use default parameters? Did the authors blast against nt or specifically against the horse genome? Which threshold did they set in order to accept the hits as horse?*

*Lines 211-213: Which software was used for mapping? Which parameters were used to ensure that the maximum proportion of mismatches was 5%?*

*Lines 213-214: It is not clear how authors went from mapping reads to assemble the mitochondrial genome. This should be described.*

*Lines 216-218: Which software used for building the consensus? How were contigs generated? Which requisites were required for a contig to be used in the assembly? (e.g. contig length, contig coverage). Which overlap between contigs was required to assemble them into a scaffold? “We built the consensus sequence of the whole mitochondrion of the Puerto Real horse with a threshold of 75% and a quality set on “Highest”. The consensus was generated out of contig pileups that represented a mean coverage of 5622.9X of the horse reference mitochondrion.”*

*Lines 229-230: “The mitochondrial data was partitioned into coding, non-coding, and RNA regions to produce a phylogeny using different model estimates for each of these regions.” What are the differences between the model estimates? Are they implemented in Geneious? What are their names?*

Reviewer #3 had several recommendations to improve the paper, particularly in terms of reproducibility and data availability. We already addressed the question of data accessibility (see above). In the methods section, we added some clarifications as requested by the reviewer as shown in the new version of the manuscript. More specifically, we added additional details on how the reads were assembled using the commercial software Geneious (lines 210-218).

- **Reviewer #4:**

*This study focused on a single ancient horse sample from 16th century /Caribbean colonial horse/. Complete mitochondrial sequencing of this single sample was performed. According to the authors, this mitochondrial genotype is associated with group A, and is the most closely related to a modern specimen of the American Chincoteague pony breed from North America (JN398377). I did not find a deposit of your genomic sequence and Genbank. Why?*

*The article is well discussed historically, but in contrast, relatively genetic research in this region is lacking. It would be good to make a comparative analysis of the available genetic data on the biodiversity of mitochondrial lines (modern and ancient) for America and the Iberian Peninsula. I would recommend adding data on modern local horses from the study region.*

Reviewer #4 also expressed some concern about the accessibility of the sequence and the reads (see above for the response). We agree with their suggestion that adding more mitochondrial genomes of horses from the Caribbean would be helpful. However, there are few published and available mitogenomes from the Caribbean on public repositories such as Genbank or the ENA, and we used all available, relevant mitogenomes in our analysis. We hope that further work on horse heritage breeds from across the Caribbean will provide more data to fill this gap.

Again, we thank the academic editor and the reviewers for their comments and suggestions. We sincerely hope the changes in this revised version of the manuscript reflects these recommendations.

Sincerely,

Nicolas Delsol

Corresponding author
