# Supplementary material for: Analysis of the earliest complete mtDNA genome of a Caribbean colonial horse (Equus caballus) from 16th-century Haiti
Source: PLoS One. 2022 Jul 27;17(7):e0270600. doi: 10.1371/journal.pone.0270600 (PMC9328532; doi:10.1371/journal.pone.0270600)
Supplement: Attachment — Submitted filename: PLOS1 rebuttal letter#2_ND_2nd resubmission.docx [file pone.0270600.s006.docx]

Dr. Nicolas Delsol

Postdoctoral Researcher,

Florida Museum of Natural History,

Dickinson Hall, 1659 Museum Road

Gainesville, FL 32611-7800

USA

[ndelsol@ufl.edu](mailto:ndelsol@ufl.edu)

Gainesville, May 25, 2022

To the PLOS One editing committee,

We would like to renew our thanks to the academic editor and the reviewers for their thoughtful comments which have greatly helped improve our manuscript. We were happy to see that two of the three reviewers found our prior submission satisfactory. Please find here our responses to the points raised by Reviewer #4 in this version. For clarity purposes, these comments are inserted in *blue italic* font before our responses.

*I did not find a deposit of your genomic sequence and Genbank?*

As stated in the letter attached to our first resubmission, the assembly of the horse mitogenome has been submitted successfully to Genbank (accession number ON168403) and the raw reads were submitted to the Sequence Read Archive (accession number PRJNA817535). These have been explicitly called out in the latest revisions to this article (lines 245-247). As customary, we have embargoed the release of these data until after the publication of the manuscript. As a proof of these submissions, we provide the confirmation emails we received from both repositories.

*It would be good to make a comparative analysis of the available genetic data on the biodiversity of mitochondrial lines (modern and ancient) for America and the Iberian Peninsula. I would recommend adding data on modern local horses from the study region.*

We agree with Reviewer 4 that such a comparative analysis of this 16^th^-century mitogenome with modern and ancient mtDNA from archaeological and modern horses from Iberia and the Americas would be extremely interesting. In the introduction section, we mention an older study that outlined some genetic similarities between American and Iberian horses using short regions of the D-loop between 360- to 442-bp long (Luís C, Bastos-Silveira C, Cothran EG, Oom M do M. Iberian Origins of New World Horse Breeds. J Hered. 2006 Mar 1;97(2):107–13).

To address Reviewer 4’s concerns, we have further clarified our approach and have explicitly explained the value of using complete mtDNA only (lines 126-128 of the revised manuscript). We also added the following paragraph at the beginning of the Results section (lines 268-275).

“This study instead explicitly focuses on the whole mitochondrial sequence following more recent horse phylogeographic analyses showing that the short segments of the mtDNA used in earlier studies were often accompanied by high levels of recurrent mutations, thus blurring the structure of the phylogenetic analyses and rendering some clades more difficult to distinguish from one another (23). Thus, most complete mitogenomes published on Genbank stem from the Achilli and colleagues study mentioned above. A GenBank query using the terms “horse mitochondrion” returned a total of 511 complete mtDNA sequences (~16.6 kbp). None of the returned sequences were individuals from Iberia and all the specimens from the Americas found using this query were included in our study.”

*In addition, the article is not written according to the requirements of PlosOne. As an example, too short a description of the results and the discussion. The abstract section is not formatted according to the requirements of the journal.*

Thank you for pointing out the formatting requirements for this journal. In this revision we have made every effort to ensure our formatting falls within the guidelines found on <https://journals.plos.org/plosone/s/submission-guidelines>. We have also refined the text in several places. We have expanded the results section, but feel that the discussion provides sufficient content to contextualize our findings.

*The Materials and Methods section describes the isolation of ancient DNA that does not conform to the protocols of good practice for working with ancient DNA.*

*Тhere are no control reactions for DNA contamination as example.*

*I cannot believe that DNA isolation is possible in the described way to dissolve 500 mg of dry bone material in 1 ml of buffer.*

Our methods for ancient DNA work have been outlined in a number of publications (Oswald et al, 2019, 2020, 2021). We have further described the Florida Museum Ancient DNA Laboratory and its protocols. As stated in our earlier MS and clarified in this version, this study did include negative controls. As per the Florida Museum aDNA laboratory, all extractions (for this study and others) include negative controls. As shown by our results in these several studies, our ratio of sample to buffer is appropriate and effective. The protocols used for DNA extraction are based not only on our work but also on published studies such as those of Yang et al. 1998, 2008 and others.

1. Yang, D. Y., Eng, B., Waye, J. S., Dudar, J. C. & Saunders, S. R. Technical note: Improved DNA extraction from ancient bones using silica-based spin columns. Am. J. Phys. Anthropol. 105, 539–43 (1998).
2. Yang, D. Y., Liu, L., Chen, X. & Speller, C. F. Wild or domesticated: DNA analysis of ancient water buffalo remains from north China. J. Archaeol. Sci. 35, 2778–2785 (2008).

To clarify our protocols and further describe the measures we used to limit cross-contamination, we have added details about our negative control reactions during the extraction process (lines 205-207).

Again, we thank the academic editor and the reviewers for their comments and suggestions. We hope the changes we have made in this revised version of the manuscript effectively addresses these recommendations.

Sincerely,

Nicolas Delsol

Corresponding author
